# Modulation of SOCS protein expression influences the interferon responsiveness of human melanoma cells

**DOI:** 10.1186/1471-2407-10-142

**Published:** 2010-04-14

**Authors:** Gregory B Lesinski, Jason M Zimmerer, Melanie Kreiner, John Trefry, Matthew A Bill, Gregory S Young, Brian Becknell, William E Carson

**Affiliations:** 1 Department of Internal Medicine, Arthur G. James Cancer Hospital and Richard J. Solove Research Institute, The Ohio State University, Columbus, OH 43210, USA; 2 Department of Surgery Arthur G. James Cancer Hospital and Richard J. Solove Research Institute, The Ohio State University, Columbus, OH 43210, USA; 3 The Center for Biostatistics, Arthur G. James Cancer Hospital and Richard J. Solove Research Institute, The Ohio State University, Columbus, OH 43210, USA; 4 Department of Molecular Virology, Immunology and Medical Genetics, Arthur G. James Cancer Hospital and Richard J. Solove Research Institute, The Ohio State University, Columbus, OH 43210, USA

## Abstract

**Background:**

Endogenously produced interferons can regulate the growth of melanoma cells and are administered exogenously as therapeutic agents to patients with advanced cancer. We investigated the role of negative regulators of interferon signaling known as suppressors of cytokine signaling (SOCS) in mediating interferon-resistance in human melanoma cells.

**Methods:**

Basal and interferon-alpha (IFN-α) or interferon-gamma (IFN-γ)-induced expression of SOCS1 and SOCS3 proteins was evaluated by immunoblot analysis in a panel of n = 10 metastatic human melanoma cell lines, in human embryonic melanocytes (HEM), and radial or vertical growth phase melanoma cells. Over-expression of SOCS1 and SOCS3 proteins in melanoma cells was achieved using the PINCO retroviral vector, while siRNA were used to inhibit SOCS1 and SOCS3 expression. Tyr^701^-phosphorylated STAT1 (P-STAT1) was measured by intracellular flow cytometry and IFN-stimulated gene expression was measured by Real Time PCR.

**Results:**

SOCS1 and SOCS3 proteins were expressed at basal levels in melanocytes and in all melanoma cell lines examined. Expression of the SOCS1 and SOCS3 proteins was also enhanced following stimulation of a subset of cell lines with IFN-α or IFN-γ. Over-expression of SOCS proteins in melanoma cell lines led to significant inhibition of Tyr^701^-phosphorylated STAT1 (P-STAT1) and gene expression following stimulation with IFN-α (IFIT2, OAS-1, ISG-15) or IFN-γ (IRF1). Conversely, siRNA inhibition of SOCS1 and SOCS3 expression in melanoma cells enhanced their responsiveness to interferon stimulation.

**Conclusions:**

These data demonstrate that SOCS proteins are expressed in human melanoma cell lines and their modulation can influence the responsiveness of melanoma cells to IFN-α and IFN-γ.

## Background

Endogenously produced cytokines of the type I and type II interferon families are critical for the recognition of developing tumors by the immune system [1]. Recent evidence has demonstrated that the actions of endogenous type I interferons (e.g. IFN-α, IFN-β) are essential for the immune surveillance of tumors by their direct actions on host immune cells [2]. Interferon-gamma (IFN-γ), a type II interferon, has also been shown to act directly upon malignant cells and thereby render them more immunogenic [3].

In addition to the role of endogenous IFNs in regulating tumor growth, IFN-α is administered to patients with metastatic disease. It is presently the only therapy approved for use as an adjuvant following surgical resection of high-risk melanoma lesions [4-8]. There is evidence that the immunostimulatory effects of IFN-α contribute to its anti-tumor activity [9-11] but exogenous IFN-α can also exert direct anti-proliferative, anti-angiogenic and pro-apoptotic effects on melanoma cells [12-15]. The predominant signal transduction pathway activated in response to both IFN-α and IFN-γ is the Janus kinase-signal transducer and activator of transcription (Jak-STAT) pathway (Reviewed in [16,17]. Our group has previously demonstrated that IFN-α induced Jak-STAT signal transduction within melanoma cells is highly variable, and, in some cases, significantly attenuated [18]. Interestingly, the expression of key signaling proteins important for IFN-α-responsiveness was intact in these IFN-resistant melanoma cells, suggesting that negative regulatory pathways for IFN-induced signal transduction might be operative.

One such negative regulatory pathway is a class of proteins called the suppressors of cytokine signaling (SOCS). The SOCS proteins consist of eight structurally related family members, SOCS1-7 and CIS (cytokine-inducible SH2-containing protein). These proteins contain a central Src-homology 2 (SH2) domain and a conserved C-terminal domain termed the SOCS box [19]. SOCS proteins can inhibit cytokine-induced signal transduction (Reviewed in [20] by multiple mechanisms including: 1) binding to phosphorylated tyrosine residues; 2) blocking access of transcription factors to their receptor sites; or 3) SOCS box-targeting of bound proteins for proteasomal degradation [21].

Expression of SOCS1 and SOCS3 has been reported in melanoma cell lines and in surgical specimens obtained from malignant melanoma patients where it indicates a poor-prognosis [22]. However, the functional effects of SOCS expression on the response of human melanoma cells to interferons has only been evaluated in a limited number of studies [23-25]. We hypothesized that SOCS1 and SOCS3 proteins may down-regulate the biological response of melanoma cells to endogenous or exogenously administered interferons.

The present study demonstrates that SOCS1 and SOCS3 proteins were expressed in a panel of melanoma cell lines from various stages of disease and in melanocytes. IFN-α and IFN-γ treatment led to further increases SOCS1 and SOCS3 expression in some human melanoma cell lines. Furthermore, over-expression of SOCS1 and SOCS3 expression led to a significant reduction in IFN-induced STAT1 phosphorylation and gene expression. Conversely siRNA-mediated inhibition of SOCS1 and SOCS3 expression enhanced the interferon-responsiveness of human melanoma cells. These data provide additional evidence that SOCS proteins regulate the direct actions of interferons on melanoma cells.

## Methods

### Reagents and Cell Lines

Recombinant human (hu) IFN-α2b (specific activity of 2 × 10^8 ^IU/mg) was purchased from Schering-Plough, Inc. (Kenilworth, NJ). The HT144, Hs294T, SK-MEL-5 and A375 human melanoma cell lines were purchased from the American Type Culture Collection (Manassass, VA). The 1106 MEL, 18105 MEL, MEL-39, 1174 MEL, FO1 and 1259 MEL human melanoma cell lines were obtained from Dr. Soldano Ferrone (University of Pittsburgh Cancer Institute, Pittsburgh, PA). The radial growth phase WM 1552c and vertical growth phase WM 793b human melanoma cell lines were provided by Dr. M. Herlyn (Wistar Institute, Philadelphia, PA) and cultured as described [26]. Human embryonic melanocytes were purchased from ScienCell Research Laboratories (Carlsbad, CA) and cultured per manufacturer's recommendations. All studies were conducted with the approval of The Ohio State University Institutional Biosafety Committee.

### Immunoblot Analysis

Expression of SOCS1 and SOCS3 proteins in melanoma cell lines was confirmed via immunoblot analysis with antibodies directed against SOCS1, SOCS3 (Abcam, Cambridge, UK), phosphorylated STAT3 (Tyr705), STAT3 (Cell Signaling Technology, Inc., Danvers, MA), or β-actin as previously described [27].

### Flow Cytometric Analysis of Phosphorylated STAT1 (P-STAT1)

Phosphorylation of STAT1 at Tyr^701 ^was measured using an intracellular flow cytometric assay as previously described; with modifications [28]. To measure STAT1 phosphorylation in retroviral-transduced cells, a goat anti-rabbit APC-conjugated secondary Ab was employed (Molecular Probes, Eugene, OR). Amplified fluorescence signals were expressed as specific fluorescence (Fsp = F_t _-F_b_), where F_t _represents the median value of total staining, and F_b _represents the median value of background staining (obtained by staining with the isotype control Ab) [28].

### Real Time RT-PCR

Real-time reverse transcription-polymerase chain reaction (RT-PCR) was used to quantitate transcript levels of the IFN-α stimulated genes ISG-15 and IFIT2 or IFN-γ-stimulated gene, IRF1 in melanoma cell cultures as previously described [28,29]. Briefly, melanoma cells were treated with IFN-α (10^4 ^U/mL), IFN-γ (1 ng/mL) or PBS for 4 hours. Total RNA was then isolated using the RNeasy RNA Isolation Kit (Qiagen, Valencia, CA), quantitated, and converted to cDNA using random hexamers as primers for first-strand synthesis. Two microliters of the resulting cDNA was used as a template to measure the levels of mRNA for ISG expression by Real-time RT-PCR with pre-designed primer/probe sets (Assays On Demand; Applied Biosystems, Foster City, CA) and 2× TaqMan Universal PCR Master Mix (Applied Biosystems) per the manufacturer's recommendations. Pre-designed primer/probe sets for human β-actin were used as an internal control in each reaction well (Applied Biosystems) and data was analyzed using Sequence Detector software (version 1.6; Applied Biosystems).

### SOCS Over-expressing Retroviral Constructs

The PINCO retroviral transfer plasmid, originally from the laboratory of Dr. P.G. Pelicci (Grignani et al., 1998), was obtained through the courtesy of Dr. Martin Sattler (Dana Farber Cancer Institute, Boston, MA). This retroviral vector permits the expression of a gene of interest from the 5' long terminal repeat (LTR) as well as the enhanced green fluorescent protein (EGFP) from an internal cytomegalovirus (CMV) immediate early promoter. The development of PINCO-based retroviral constructs that over-express SOCS1 and SOCS3 proteins has been previously described by our group [30,31]. SOCS1-, and SOCS3-overexpressing retroviral supernatants were generated by transient transfection of the Phoneix-Ampho packaging cell line with appropriate PINCO-SOCS expression plasmid DNA and 0.9 μg of a plasmid DNA expressing the VSV-G protein (pVSV-G) using the ProFection^® ^Mammalian Transfection System-Calcium Phosphate kit according to manufacturer's instructions (Promega, Madison, WI) as previously described [31]. Human melanoma cell lines (HT144, 1259 MEL) were transduced with the resulting SOCS-over-expressing retroviral constructs as previously described [31].

### Inhibition of SOCS1 and SOCS3 Using Small Inhibitory RNA

Silencing of SOCS1 and SOCS3 expression was accomplished using pSilencer siRNA expression vectors (Invitrogen). siRNA oligonucleotides that target SOCS1 (5'-CTGGTTGTTGTAGCAGCTTAA-3') and SOCS3 (5'-TCGGGAGTTCCTGGACCAGTA-3') sequences were cloned into the pSilencer vector per manufacturer's recommendations. Briefly, 1259 MEL melanoma cells were transfected with 2 μg siRNA encoding plasmids or pSilencer vector alone (negative control) via electroporation using the Amaxa Nucelofector device and cell-specific nucleofector reagent (Amaxa, Inc., Gaithersburg, MD) according to the manufacturer's recommendations.

### Statistical Methods

Mixed effects regression models were used to analyze the experimental outcomes, including a random effect for each experiment. The outcomes of specific fluorescence and fold change in gene expression were both log-transformed to meet the necessary model assumptions of normality and constant variance. A Tukey-Kramer adjustment for multiple comparisons was used to calculate the p-values when comparing groups within the same experiment [32]. Adjusted p-values less than 0.05 were considered statistically significant.

## Results

### Melanoma Cell Lines Express SOCS1 and SOCS3 Proteins

**Figure 1:**
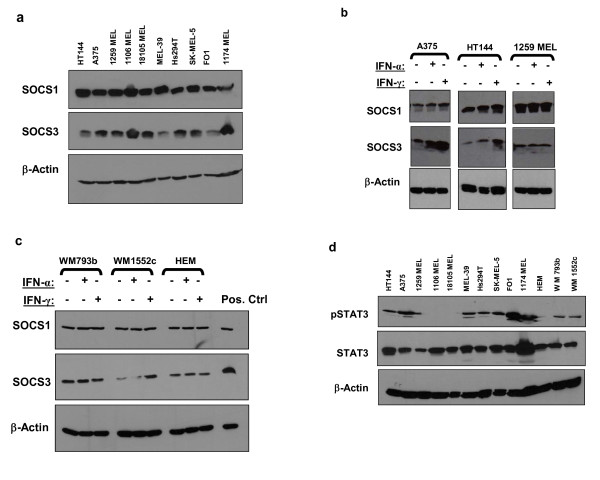
**SOCS1, SOCS3 and STAT3 expression in melanoma cell lines**. **(a) **Basal expression of SOCS1 and SOCS3 proteins in a panel of n = 10 human melanoma cell lines was measured by immunoblot analysis. β-actin served as a control for equal loading. Basal and IFN-induced SOCS1 and SOCS3 expression was evaluated in **(b) **human metastatic melanoma cell lines and **(c) **human embryonic melanocytes (HEM) or vertical (WM793b) and radial (WM1552c) human melanoma cell lines by immunoblot following treatment of human melanoma cell lines with IFN-α (4 hours; 10^4^U/mL) and IFN-γ (4 hours; 10 ng/mL). A375 cells were used as a positive control for SOCS1 and SOCS3 expression (Pos. Ctrl). Immunoblot data shown are representative from at least 3 independent experiments. **(d) **Constitutive phosphorylation of STAT3 was evaluated in this panel of cell lines by immunoblot analysis. Antibodies directed against STAT3 protein and β-actin were included to control for variations in STAT3 across cell lines and equal loading, respectively.

The baseline expression of SOCS1 and SOCS3 protein was measured in a panel of n = 10 human metastatic melanoma cell lines by immunoblot analysis. These data revealed that all lines expressed both SOCS1 and SOCS3 proteins (Figure 1a). Of note, pre-incubation of primary antibody with commercially available SOCS-specific peptide competitors (Abcam, Inc.) eliminated SOCS3 specific immunoreactivity (Data not shown). Immunoblot analysis in representative cell lines was subsequently used to evaluate the effects of IFN-α and IFN-γ stimulation on SOCS1 and SOCS3 protein expression *in vitro *(Figure 1b). Immunoblot analysis revealed an induction of SOCS1 and SOCS3 proteins in some melanoma cell lines following a four hour stimulation with IFN-α (10^4 ^U/mL) or IFN-γ (10 ng/mL). The IFN-induced expression of SOCS1 and SOCS3 proteins within individual cell lines was variable. For example, IFN-α and IFN-γ induced SOCS3 protein expression in the A375 melanoma cell line while both SOCS1 and SOCS3 proteins were upregulated in the HT144 cell line (Figure 1b). In contrast, neither SOCS1 nor SOCS3 proteins were further upregulated in the 1259 MEL cell line following IFN stimulation. To determine whether SOCS protein expression might be restricted to melanoma cells at a particular stage of development, we conducted similar experiments in human embryonic melanocytes (HEM) and in the WM793b and WM1552c human melanoma cell lines. These two cell lines are derived from vertical and radial growth phase melanoma cells, respectively. SOCS1 and SOCS3 were expressed at basal levels in each of these cell types (Figure 1c). Treatment with IFN-α or IFN-γ did not upregulate SOCS1 expression in any of these cell lines, while SOCS3 was upregulated by IFN-γ only in the WM1552c radial growth phase cell line.

### Phosphorylated STAT3 expression in human melanoma cell lines

The STAT3 transcription factor promotes a metastatic phenotype and has been shown to be constitutively phosphorylated in human melanoma cell lines. Importantly, STAT3 is also sensitive to inhibition by SOCS proteins [33] and prior studies in melanoma brain metastases have suggested that loss of SOCS1 expression could promote metastasis via elevated STAT3 signaling. We therefore analyzed pSTAT3 levels in order to determine whether SOCS1 protein expression and basal STAT3 phosphorylation were associated in the panel of melanoma cell lines. These data indicated that basal STAT3 phosphorylation (at Tyr^705^) and SOCS1 expression were present concurrently (Figure 1d). These data suggest that the presence of SOCS1 does not alter the level of basal pSTAT3.

### Inhibition of IFN-Responsiveness in Melanoma Cell Lines that Over-express SOCS Constructs

**Figure 2:**
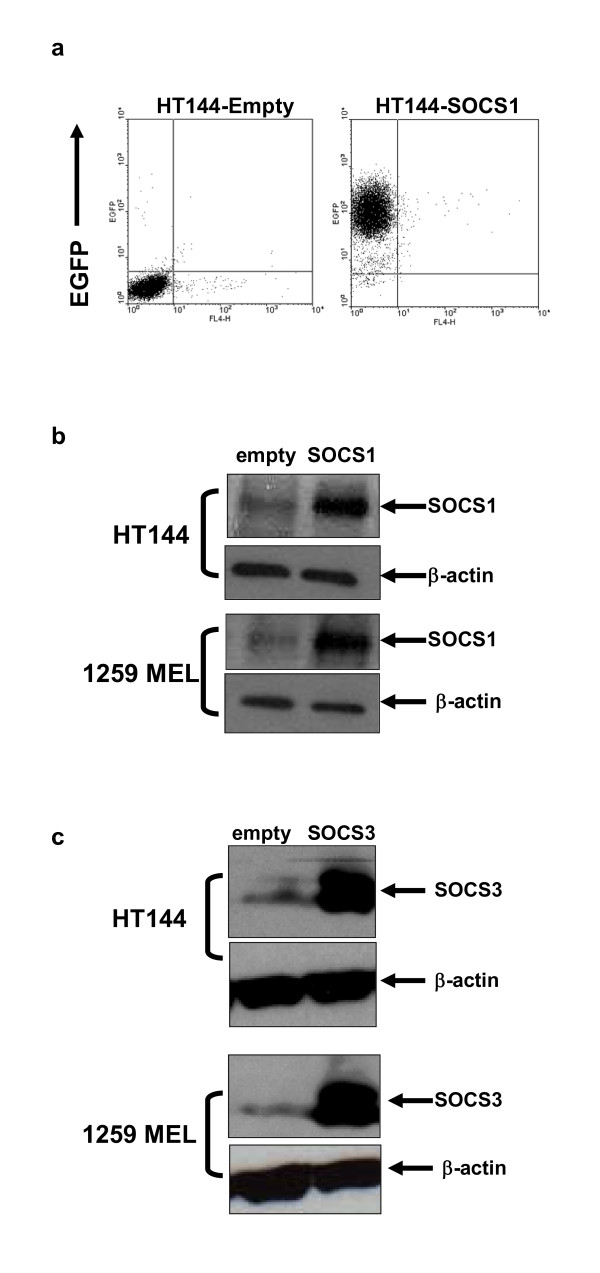
**Retroviral mediated over-expression of SOCS1 and SOCS3**. GFP expression was evaluated by flow cytometry in melanoma cells stably transduced with PINCO retroviral vectors over-expressing SOCS1 or SOCS3. A representative example is shown in **(a)**. Over-expression of **(b) **SOCS1 and **(c) **SOCS3 at the protein level was confirmed by immunoblot analysis. Membranes were stripped and re-probed with β-actin as a control for equal loading.

**Figure 3:**
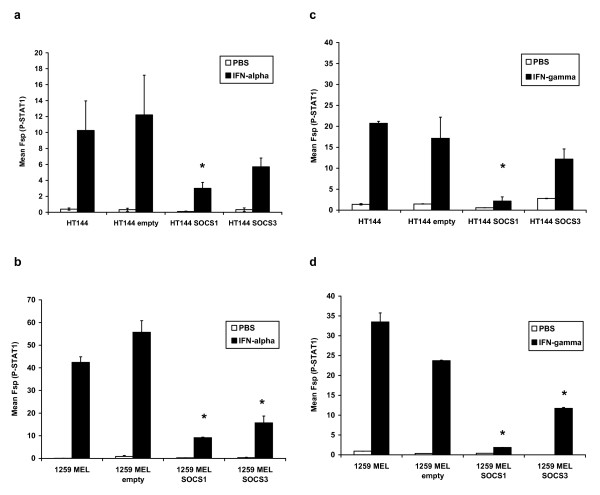
**Reduced IFN-induced P-STAT1 in melanoma cells over-expressing SOCS1 and SOCS3**. Phosphorylated STAT1 was measured by flow cytometry following a 15-minute stimulation of SOCS1- and SOCS3-over-expressing cell lines with **(a, b) **IFN-α or **(c, d) **IFN-γ. Both non-transduced and empty vector transduced melanoma cell lines served as negative controls in these experiments. Error bars represent standard deviation and are derived from triplicate experiments. * Denotes p < 0.05 as compared to empty vector transduced cells.

**Figure 4:**
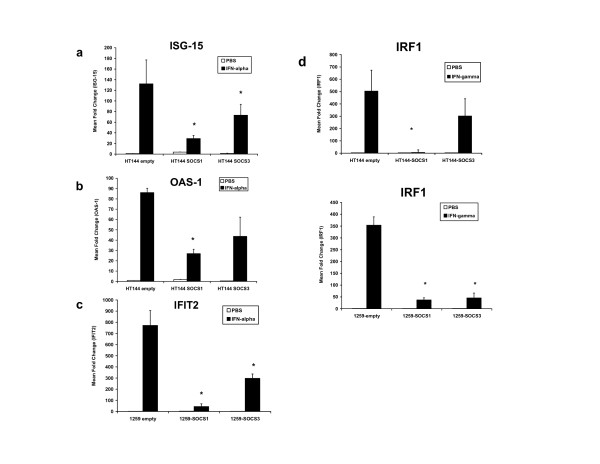
**Reduced IFN-stimulated gene expression in melanoma cells over-expressing SOCS1 and SOCS3**. Transcript levels of the IFN-α-responsive genes **(a) **ISG-15, **(b) **OAS-1, and **(c) **IFIT2 were measured following a 4 hour stimulation of SOCS1- and SOCS3-over-expressing cell lines with IFN-α (10^4 ^U/mL) by Real Time PCR. **(d) **Transcript levels of the IFN-γ-responsive gene IRF1 was also measured in the cell lines following a 4 hour stimulation with 1 ng/mL IFN-γ. All data were normalized to β-actin (housekeeping gene) and expressed relative to PBS-treated cells. * Denotes p < 0.05 as compared to empty vector transduced cells.

To further validate the role of SOCS protein expression in regulating IFN-responsiveness of representative human melanoma cell lines (HT144, 1259 MEL), we employed retroviral constructs that expressed SOCS1 and SOCS3. Non-transduced cells and cells transduced with the empty PINCO retroviral vector were used as controls in these assays. Twenty-four hours following transduction of human melanoma cell lines with the SOCS1 and SOCS3-expressing retroviral constructs or empty PINCO vector, the expression of EGFP was confirmed in each cell line by flow cytometry (Figure 2a). EGFP^+ ^cells were sorted by flow cytometry and cultured for use in subsequent experiments. Transduced melanoma cell lines over-expressed SOCS1 and SOCS3 proteins as determined by immunoblot analysis (Figure 2b-c). No increase in SOCS1 or SOCS3 expression was observed in any melanoma cell line transduced with the empty PINCO vector. Analysis of STAT1 phosphorylation by flow cytometry indicated that IFN-α-induced signal transduction was significantly inhibited in HT144 and 1259 MEL cells that over-expressed SOCS1 as compared to either parental cells or those transduced with the empty PINCO vector (all p-values = 0.019). Over-expression of SOCS3 led to a significant reduction in IFN-α-induced signal transduction in the 1259 MEL cell line (p = 0.0052), but this did not reach statistical significance in the HT144 cell line (Figure 3a-b; p = 0.087). Similar results were obtained for IFN-γ-induced activation of STAT1 (Figure 3c-d). IFN-α and IFN-γ responsiveness was most inhibited by over-expression of SOCS1 as compared to SOCS3. As expected, SOCS1 over-expression significantly reduced the transcription of IFN-α regulated genes (ISG-15 and OAS1) and the IFN-γ induced gene IRF1 in these cell lines (Figure 4a-d; all p-values ≤ 0.01). Similar to our signal transduction data, over-expression of SOCS3 in the 1259 MEL cell line produced a statistically significant inhibition of IFN-stimulated gene expression (all p-values ≤ 0.0049) but results for HT144 were mixed. A significant difference was observed for expression of ISG-15 in the HT144 cell line (p = 0.0144), but not for OAS-1 (p = 0.0511) or IRF1 (p = 0.49).

### Interferon-Responsiveness in Melanoma Cell Lines Transfected With SOCS1 or SOCS3-targeted siRNA constructs

**Figure 5:**
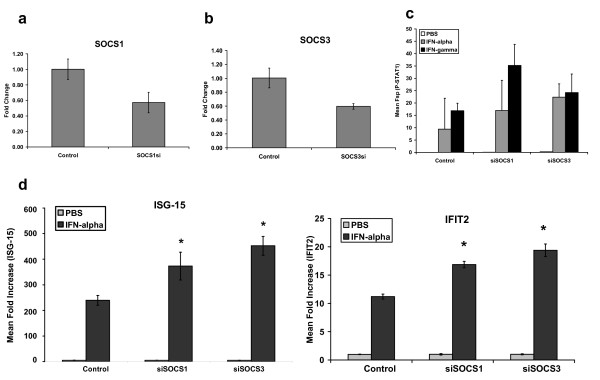
**siRNA-mediated reduction of SOCS1 and SOCS3 enhances IFN-responsiveness of melanoma cells**. Real Time PCR was used to validate reduced expression of **(a) **SOCS1 and **(b) **SOCS3 48 hours following transient transfection of 1259 MEL cells with empty pSilencer vector (Control) or pSilencer expressing SOCS-specific siRNA. Data were normalized to β-actin (housekeeping gene) and expressed as mean expression values relative to cells transfected with control vector. **(c) **P-STAT1 levels were measured following a 15 minute stimulation of melanoma cells with IFN-α (10^4 ^U/mL) or IFN-γ (1 ng/mL) by flow cytometry. PBS (vehicle) treated cells served as negative controls. Data represent the mean Fsp values (± standard deviation) from n = 2 experiments with similar results. **(d) **IFN-stimulated gene expression was evaluated in response to a 4 hour treatment with IFN-α (10^4 ^U/mL) 48 hours post-transfection with empty vector (negative control) or vector expressing SOCS1- or SOCS3-specific siRNA. Data were normalized to β-actin and expressed relative to PBS-treated cells. * Denotes p < 0.05 as compared to empty vector transfected cells.

The interferon-responsiveness of SOCS1 and SOCS3-positive 1259 MEL melanoma cells transfected with a vector expressing siRNA constructs targeting SOCS1 or SOCS3 was next investigated. Cells transfected with an empty vector served as negative controls in these studies. Transfection efficiency was typically > 90% as determined by transfection of cells with a GFP-expressing plasmid in parallel (data not shown). Reduced levels of SOCS1 and SOCS3 transcript were confirmed by Real Time PCR (Figure 5a-b). We proceeded to test whether the reduced level of SOCS expression would affect the IFN-responsiveness of melanoma cells. Due to high basal expression, complete knockdown of SOCS1 and SOCS3 in these cells was difficult to achieve. However, their reduced expression following transfection of 1259 MEL cells with SOCS1 or SOCS3 siRNA led to consistent increases in IFN-α or IFN-γ-induced P-STAT1 as compared to cells transfected with control siRNA (Figure 5c). Transcription of IFN-α-induced genes (IFIT2, ISG-15) was tested by Real Time PCR and found to be significantly increased in cells transfected with SOCS1siRNA or SOCS3siRNA (Figure 5d; all p-values < 0.005).

## Discussion

In the present study we have demonstrated that human melanoma cell lines express basal levels of SOCS1 and SOCS3 and that these proteins are further increased upon stimulation with type I and type II IFNs. Over-expression of SOCS1 and SOCS3 proteins in melanoma cell lines led to a significant decrease in the IFN-responsiveness of melanoma cells. Conversely, siRNA-mediated reduction of SOCS1 and SOCS3 led to an increase in IFN-responsiveness. These data support a role for basal expression of SOCS1 and SOCS3 as contributors to IFN-resistance in human melanoma cells.

Maximizing the direct effects of type I and type II IFNs on melanoma tumors is a challenge due to the genetic heterogeneity of these cell types. Indeed, defects in key components of IFN-α induced signal transduction pathways have been noted in several malignant melanoma cell lines. For example, tumor cell lines with defects in STAT1, IRF9 and Jak1 have been identified and have been found to have reduced *in vitro *responsiveness to IFN-α [14,34,35]. Previous reports from our group and others have also suggested that the direct actions of IFN-α on melanoma cells are highly variable and often attenuated, even when the expression of Jak-STAT intermediates were intact [18,36,37]. Data from the present study extends these initial observations and further suggests that expression of SOCS proteins represent a means by which melanoma cells can achieve IFN-resistance.

In agreement with the present study, Li *et al*. have shown that SOCS1 and SOCS3 proteins were expressed in both human melanoma cell lines and primary tumors from melanoma patients. In this prior study, SOCS1 expression in primary tumors from melanoma patients was thought to be an indicator of poor prognosis as it correlated with Breslow thickness and Clark's Level [22]. Although the effect of SOCS1 or other SOCS family members (i.e. SOCS3) on IFN-α-responsiveness of melanoma cells was not evaluated in this prior study, our observations suggest that SOCS1 and SOCS3 could be a clinically relevant inhibitor of cytokine action.

In contrast to data from our group and others [38], Kovarik *et al*. did not observe basal or IFN-α-induced SOCS3 expression in human melanoma [25]. These differences could simply reflect the fact that unique panels of human melanoma cell lines were used in the two separate reports. Importantly, the majority of cell lines used in the present study were derived from metastatic melanoma lesions, while the study by Kovarik *et al*. utilized many less-aggressive, radial-growth phase cell lines derived from primary melanomas. The lack of IFN-α-induced SOCS3 expression in the study by Kovarik *et al*. could also be attributed to the fact that a majority of their data was obtained following only a 30 minute stimulation with IFN-α *in vitro*, and longer (4 hour) IFN-α stimulation was only reported in two cell lines [25]. Together, these data suggest that SOCS3 expression may be associated with more metastatic or aggressive melanoma tumors. In support of this argument is another observation that prolonged cultivation of melanoma cells has been reported to lead to an increase in the constitutive expression of SOCS3 transcripts *in vitro *[23]. Finally, and in agreement with our data, studies conducted by other groups have also shown that basal SOCS3 expression was detectable at the transcript and protein level in the metastatic A375 melanoma cell line [24]. Together these data highlight the phenotypic heterogeneity of melanoma and indicate the variable biologic responsiveness of this problematic malignancy.

A role for SOCS proteins in tumor resistance to cytokines has also been suggested in the setting of hematologic malignancy. For example, Sakai *et al*. demonstrated that constitutive expression of SOCS3 affected the IFN-α sensitivity of chronic myelogenous leukemia (CML) cell lines and blast cells from patients in CML blast crisis [39], while Brender *et al*. demonstrated that SOCS3 protected T cell lymphoma cells against growth inhibition by IFN-α [40]. Other reports have demonstrated that silencing of SOCS1 can enhance the anti-tumor activity of type I or type II IFNs by regulating apoptosis in neuroendocrine tumor cells [41]. Silencing of SOCS1 also enhanced the anti-proliferative effects of IFNs on the murine B16 and Colon26 cell lines [42]. The present study provides further evidence for the ability of SOCS proteins to regulate the IFN-responsiveness of melanoma cells.

In the present study, over-expression of SOCS1 significantly inhibited IFN-responsiveness of melanoma cells and conversely siRNA-mediated reduction of SOCS1 enhanced the IFN-response. Based on these results alone, the relative role each individual SOCS protein plays in regulating the IFN-response in melanoma cells remains unclear and is likely variable between patients. Furthermore, the profile of other negative regulatory proteins is complex, and in theory could compensate for, or synergize with existing SOCS proteins to limit cytokine responsiveness in the cell. An intriguing study by Huang *et al*. highlights the controversial role of SOCS1 protein in the setting of melanoma. In this report, the authors used an experimental model of A375 melanoma that metastasizes to the brain and demonstrated that SOCS1 expression is significantly reduced in brain metastases as compared to the original tumor [43]. The authors later show that this loss of SOCS1 expression is a critical event leading to elevated STAT3 signaling and over-expression of factors that promote cellular invasion and angiogenesis. These data caution that modulating SOCS1 expression as a therapeutic strategy also has the potential to promote metastasis via STAT3 and this possibility should be carefully investigated in pre-clinical studies.

The inhibition of negative regulatory pathways to enhance the anti-tumor effects of cytokines represents a potentially novel approach against malignancy. Prior observations have primarily focused on inhibition of SOCS proteins in immune cells to allow for a greater anti-tumor effect. For example, Shen et al. have demonstrated that silencing of SOCS1 by siRNA in dendritic cells used as a therapeutic vaccine strategy resulted in superior anti-tumor activity in a murine B16F10 model of melanoma [44]. Our laboratory has also demonstrated that exogenously administered IFN-α induced profound in vivo anti-tumor activity that was immune-mediated (via CD8+ T cells) in SOCS1 and SOCS3 deficient mice [31]. Data from the present study have expanded our understanding of SOCS protein expression in melanoma and suggest that SOCS1 and SOCS3 proteins within the tumoral compartment represent a potential target that deserves investigation in future pre-clinical studies.

## Conclusions

The present data suggest that SOCS1 and SOCS3 proteins represent a means by which melanoma cells can evade the direct effects of IFN-α and IFN-γ. The role for intratumoral SOCS1 and SOCS3 proteins as novel therapeutic targets remains deserves further evaluation in pre-clinical studies of melanoma or other malignancies.

## Competing interests

The authors declare that they have no competing interests.

## Authors' contributions

GBL wrote manuscript, designed experiments, performed experiments. JMZ performed experiments with construction of retroviral vectors. MK performed flow cytometry, Real Time PCR experiments. JT performed immunoblot, flow cytometry and Real Time PCR experiments.

MAB performed immunoblot experiments. GSY performed statistical analysis, wrote manuscript. BB designed and constructed retroviral vectors. WEC wrote manuscript designed experiments. All authors read and approved the final manuscript.

## Pre-publication history

The pre-publication history for this paper can be accessed here:

http://www.biomedcentral.com/1471-2407/10/142/prepub
